# Necrotizing Enterocolitis Secondary to Congenital Syphilis in an 18‐Day‐Old Neonate: Successful Treatment With Intravenous Penicillin

**DOI:** 10.1155/crdi/6360379

**Published:** 2026-10-21

**Authors:** Chariya Chap, Vissotsak Bin, Sakviseth Bin

**Affiliations:** ^1^ Neonatal Ward National Pediatric Hospital Phnom Penh Cambodia; ^2^ University of Health Sciences Phnom Penh Cambodia akdeniz.edu.tr; ^3^ Neonatal Intensive Care Unit Calmette Hospital Phnom Penh Cambodia akdeniz.edu.tr

**Keywords:** congenital syphilis, necrotizing enterocolitis, neonate, *Treponema pallidum*, vertical transmission

## Abstract

Congenital syphilis remains a preventable yet persistent global health challenge, particularly in resource‐limited countries. Gastrointestinal involvement is uncommon, and necrotizing enterocolitis (NEC) has rarely been reported as a manifestation of early congenital syphilis. We present the case of an 18‐day‐old neonate who was initially admitted with a diagnosis of NEC, with abdominal distension, feeding intolerance, and characteristic radiologic signs, corresponding to Grade IIb NEC according to the Modified Bell criteria. However, the presence of desquamation on the soles, along with a markedly enlarged liver, warranted a test for congenital syphilis, which was subsequently confirmed. The infant was successfully treated with intravenous penicillin alongside standard medical therapy for NEC, resulting in clinical recovery without surgical intervention. This case underscores the role of *Treponema pallidum* as a potential, yet often unrecognized, causative agent of NEC in neonates. Early recognition and timely, targeted antibiotic therapy are critical to preventing severe complications.

## 1. Introduction

Congenital syphilis, caused by *Treponema pallidum*, is vertically transmitted; its complications can be peripartum such as miscarriage and premature delivery and postpartum such as desquamation or pemphigus [1, 2]. Compared with hepatosplenomegaly, hepatitis, and cholestasis, necrotizing enterocolitis (NEC) is rare in the case of early congenital syphilis [1–3]. The exact mechanism by which *the spirochete* may contribute to intestinal injury has not been clearly elucidated. However, hyperechogenic bowel observed in fetal infection, though rare, may reflect underlying intestinal inflammation or ischemia, suggesting a predisposition to postnatal bowel injury [4, 5]. In what follows, we report a rare case of NEC in a neonate diagnosed with congenital syphilis at the age of 18 days, highlighting the diagnostic and therapeutic challenges encountered in a resource‐limited setting in Cambodia.

## 2. Case Presentation

An 18‐day‐old male infant was referred and admitted to the neonatology department with a 2‐day history of abdominal distension, vomiting, and fever. He was born vaginally at 39 weeks of gestation, with a birth weight of 3000 g and good adaptation at birth, at a provincial hospital. The mother, a secundigravida, had had a history of miscarriage at approximately 20 weeks of gestation during her first pregnancy due to an unknown cause. As for this pregnancy, on the other hand, she had received only one antenatal care (ANC) visit from the hospital due to financial constraints and transportation barriers. There were no records of maternal serologic testing.

As shown by the timeline (Figure 1), on arrival, the infant appeared sick, pale, and apathetic, with no weight gain. On examination, he presented with bloated abdominal (abdominal circumference of 40 cm), abundant green gastric fluid, and hepatomegaly (liver palpable 5 cm below the costal margin); therefore, the presumptive diagnosis was NEC in a term infant with postnatal growth failure. Abdominal radiography revealed diffuse gaseous distension of bowel loops and bowel wall thickening. No pneumatosis intestinalis or pneumoperitoneum was identified (Figure 2). Laboratory results showed leukocytosis (22.9 × 10^9^/L) with neutrophil predominance (11.9 × 10^9^/L), moderate thrombocytopenia (82 × 10^9^/L), mild anemia (hemoglobin 11.9 g/dL), elevated C‐reactive protein (CRP) (32 mg/L), and mild hyponatremia (130 mmol/L) (Table 1). These findings were consistent with grade IIb NEC according to the modified Bell criteria. Following consultation with a pediatric surgeon, the infant was managed medically with close monitoring (Table 2). After discontinuing feeding, a gastric tube was inserted for decompression, along with parenteral nutrition and broad‐spectrum intravenous antibiotics (ceftriaxone, metronidazole, and gentamicin), in accordance with the national guideline.

**FIGURE 1:**
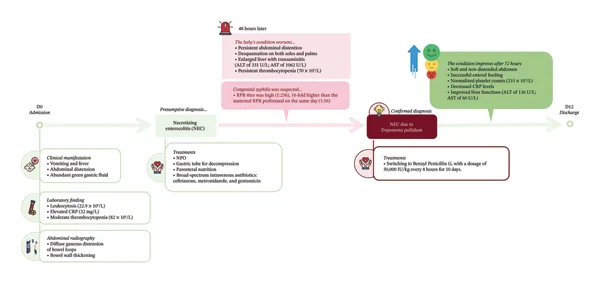
Timeline of our case report.

**FIGURE 2:**
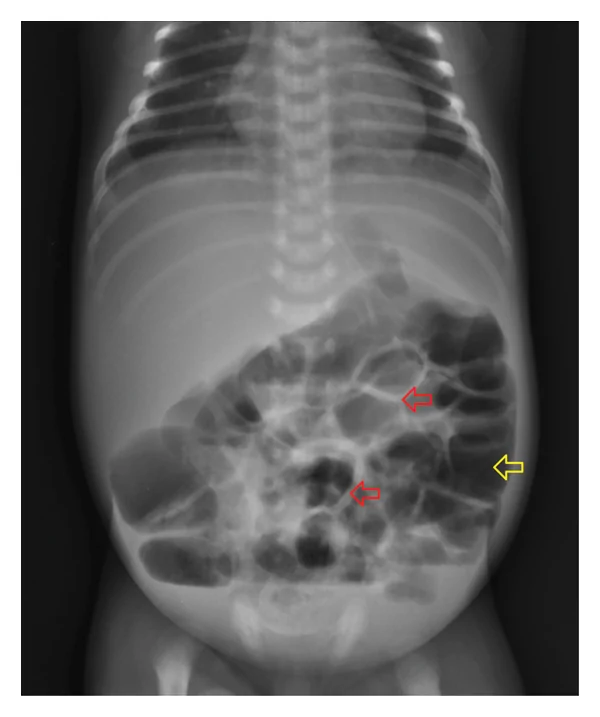
Abdominal radiography showing dilated loops (yellow arrow), with bowel wall thickening (red arrow).

**TABLE 1:** Laboratory investigation during hospitalization.

	Day 1	Day 3	Day 6	Day 10
		Penicillin *G* day 1	Penicillin *G* day 4	
White blood cell (× 10^9^/L)	22.9	23.5	14.3	17.5
Platelets (× 10^9^/L)	82	70	233	291
Hemoglobin (g/dL)	11.9	9.2	9.5	8.2
C‐reactive protein (mg/L)	32	24.12	12.14	0.17
Aspartate aminotransferase (U/L)		1062	60	
Alanine transaminase (U/L)		331	136	
TPHA		Reactive		
Rapid plasma reagin (RPR)		RPR 1:256		
Hemoculture	Neg			

Abbreviations: RPR, rapid plasma reagin; TPHA, *Treponema pallidum* hemagglutination assay.

**TABLE 2:** Modified Bell’s severity staging criteria for NEC in neonates [6].

Stage	Classification of NEC	Systemic signs	Abdominal signs	Radiographic sign
IA	Suspected	Temperature instability, apnea, bradycardia, lethargy	Gastric retention, abdominal distention, emesis, heme‐positive stool	Normal or mild intestinal dilation, mild ileus
IB	Suspected	Same as above	Grossly bloody stool	Same as above
IIA	Definite, mildly ill	Same as above	Same as above, plus absent bowel sounds with or without abdominal tenderness	Intestinal dilation, ileus, pneumatosis intestinalis
IIB	Definite, moderately ill	Same as above, plus mild metabolic acidosis and thrombocytopenia	Same as above, plus absent bowel sounds, definite tenderness, with or without abdominal cellulitis or right lower quadrant mass	Same as IIA, plus ascites
IIIA	Advanced, severely ill, intact bowel	Same as IIB, plus hypotension, bradycardia, severe apnea, combined respiratory and metabolic acidosis, DIC, and neutropenia	Same as above, plus signs of peritonitis, marked tenderness, and abdominal distention	Same as IIA, plus ascites
IIIB	Advanced, severely ill, perforated bowel	Same as IIIA	Same as IIIA	Same as above, plus pneumoperitoneum

However, after 48 h of hospitalization, the abdomen remained distended despite stable hemodynamics without any signs of peritonitis. A babygram revealed no bony abnormalities and persistent bowel loop distension with bowel wall thickening compared with the initial radiograph. Laboratory results revealed a stable white blood cell count but persistent thrombocytopenia (70 × 10^9^/L). The blood culture obtained since admission was sterile. On inspection, desquamation appeared on both soles, along with a markedly enlarged liver (Figure 3). Liver function tests showed marked transaminitis, with aspartate aminotransferase (AST) of 1062 U/L and alanine aminotransferase (ALT) of 331 U/L. Congenital syphilis was suspected, warranting serologic tests. As a result, the neonate’s RPR titer was high (1:256), 16‐fold higher than the maternal RPR performed on the same day (1:16), thus confirming our diagnosis of NEC due to *Treponema pallidum*. Benzyl Penicillin *G* was then used instead, with a dosage of 50,000 IU/kg every 8 h for 10 days.

**FIGURE 3:**
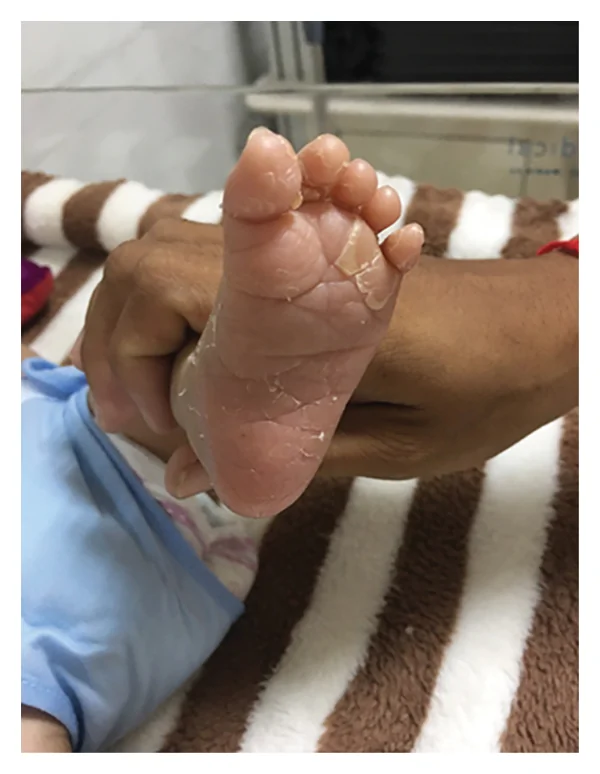
Peeling on both feet.

After the penicillin therapy was administered for 72 h, the patient showed significant clinical improvement. The abdomen became soft and less distended, and the peeling on the soles and palms improved. Laboratory parameters also improved (Table 1), with normalized leukocyte and platelet counts and decreased CRP levels. Liver function tests also improved (AST of 60 U/L and ALT of 136 U/L). Enteral feeding was successfully introduced.

After completing a 10‐day regimen of benzyl penicillin, he was discharged home with normal laboratory findings and a negative CRP result and had gained weight to 3400 g. The infant continued to gain weight appropriately, reaching 3600 g at the follow‐up (10 days after discharge).

## 3. Discussion

*Treponema pallidum* can be transmitted through the placenta from a mother to the fetus at any time during pregnancy. Untreated maternal primary or secondary syphilis is associated with a 25% risk of stillbirth, a 14% risk of neonatal death, and a 41% risk of congenital syphilis in live‐born infants [1, 7]. However, most infants (approximately 70%) born to mothers with untreated syphilis are asymptomatic at birth and may develop clinical manifestations months or even years later if they do not receive appropriate treatment [2, 8].

Congenital syphilis is classified into two categories. Early congenital syphilis, which our case focuses on, occurs typically during the first 2 years of life and presents within the first few weeks after birth with variable signs such as pemphigus syphiliticus, copper‐colored skin rashes, desquamation, organomegaly, thrombocytopenia, anemia, cataracts, bone abnormalities, and central nervous system invasion or neurosyphilis. Late congenital syphilis, on the other hand, may manifest after 2 years of age, including Hutchinson’s incisors, chorioretinitis, eighth nerve deafness, intellectual disability, and hydrocephalus. However, NEC has been rarely reported in either category [1, 2, 7–10].

NEC is a life‐threatening gastrointestinal complication in neonates. In more than 90% of cases, it typically occurs between two and 3 weeks of life, particularly in preterm infants born before 32 weeks of gestation with a very low birth weight (< 1500 g). On the contrary, approximately 10% of NEC cases occur in term infants, particularly in those with risk factors such as formula feeding, cyanotic congenital heart disease, neonatal sepsis, fetal growth restriction, and perinatal asphyxia [11–13].

NEC in preterm infants can develop in neonates with well‐tolerated feedings, contrary to term infants, in whom it typically occurs in those already hospitalized in the NICU with serious predisposing conditions such as congenital heart disease or sepsis. Despite the different underlying risk factors, the clinical manifestations in both populations are generally similar and may include bilious vomiting, abdominal distention, and rectal bleeding (hematochezia). However, in preterm infants, NEC may also present with nonspecific systemic signs such as apnea, respiratory distress, lethargy, hypothermia, and localized abdominal findings including erythema or induration [6, 14].

The diagnosis of NEC is based on characteristic clinical presentations and confirmed by abdominal imaging. Abdominal radiography may reveal dilated bowel loops, pneumatosis intestinalis (gas within the small bowel wall), portal venous gas, or signs of perforation (pneumoperitoneum) [15]. Abdominal ultrasound may also be useful due to its higher sensitivity in assessing fluid collections, bowel wall thickness, peristalsis, and perfusion; however, it is operator‐dependent and requires further evaluation [16]. Other laboratory findings can support the diagnosis and help assess the severity of NEC. Thrombocytopenia and leukopenia, predominantly neutropenia, are associated with a poor prognosis [17]. Additional abnormalities, such as persistent hyponatremia (< 130 mmol/L), hyperglycemia, and metabolic acidosis, may indicate sepsis or necrotic bowel lesions [18]. Blood culture is recommended as part of the sepsis workup when NEC is suspected; however, it may yield positive results in only 23.9%–38.5% of cases [19, 20].

The modified Bell staging criteria have been used since the 1970 s to evaluate the severity of NEC based on clinical (local and systemic), radiographic, and laboratory findings. There are three stages: Stage I (suspected cases), Stage II (definitive or proven), and Stage III (severe, advanced, or surgical) [21]. Depending on the severity stage, medical interventions include bowel decompression, discontinuation of enteral feeding, and administration of parenteral nutrition. Bowel rest is recommended for at least 24 h in case of suspected NEC (Stage I) and up to 10 days in definitive cases (Stage II). Close clinical monitoring, along with laboratory findings (leukocyte and platelet counts) and imaging (abdominal radiographs), is crucial. Broad‐spectrum intravenous antibiotics may be administered for 7–10 days in confirmed cases. A pediatric surgeon should be consulted in cases of pneumoperitoneum or worsening clinical condition. However, once surgical intervention is required, the prognosis can be poor [6].

In the existing literature, few reports have described the co‐occurrence of NEC and congenital syphilis; however, the evidence supporting a causal relationship remains limited. A two‐center retrospective study, involving 39 term neonates diagnosed with NEC and classified as Bell’s Stage II or III, identified three cases (7.7%) of congenital syphilis [22]. Çelik et al. reported a case of a preterm infant born at 29 weeks’ gestation who was initially treated for congenital syphilis and subsequently developed NEC on day 15 of life (perforated, Bell’s Stage III). Despite multiple surgical interventions, the infant died on day 54. Given that the onset of NEC occurred five days after the completion of 10‐day penicillin therapy, NEC is more likely attributable to complications of prematurity rather than a direct manifestation or complication of congenital syphilis [23]. Pugh et al. described a case of NEC occurring on day 10 of life in a term neonate (37 4/7 weeks), which coincided with the final dose of penicillin, treated for possible congenital syphilis. The authors hypothesized that the NEC was associated with antibiotic exposure, specifically penicillin, and therefore considered it an unusual adverse effect of penicillin therapy. However, the early‐term infant was formula‐fed, and both formula feeding and antibiotic exposure may have contributed to NEC, although causality remains uncertain [17].

Our case describes NEC as a rare clinical manifestation of congenital syphilis. The neonate was born to a mother with undiagnosed syphilis during pregnancy due to a lack of ANC visits. At the third week of life, the infant developed NEC, which was confirmed by clinical and radiologic findings and the unresponsiveness to standard management. However, additional clinical features, including enlarged liver and desquamation of the soles, raised suspicion for congenital syphilis, which was later confirmed by a highly elevated RPR (titer of 1:256). The infant showed a significant clinical improvement after a 10‐day course of intravenous penicillin *G*.

## 4. Conclusion

NEC is a life‐threatening neonatal gastrointestinal disorder requiring prompt and effective treatment to prevent severe surgical complications. *Treponema pallidum*, though rare, is an important associated pathogen and should be considered in neonates with NEC that is unresponsive to standard antibiotics. Congenital syphilis is preventable and treatable through appropriate maternal screening, timely diagnosis, and effective management of affected neonates [24].

## Author Contributions

All authors were involved in the writing, editing, and finalizing of this case report.

## Funding

The authors received no financial support for the research, authorship, and/or publication of this article.

## Ethics Statement

Institutional Review Board (IRB) ethical approval was not required for a case report.

## Consent

Written informed consent was obtained from the patient’s parents and is available for review upon request.

## Conflicts of Interest

The authors declare no conflicts of interest.

## Supporting Information

Additional supporting information can be found online in the Supporting Information section.

## Supporting information


**Supporting Information** CARE‐checklist‐Case NEC.

## Data Availability

Data used to support the findings of this study are available from the corresponding author upon request.
